# The Cytoscape Automation app article collection

**DOI:** 10.12688/f1000research.15355.1

**Published:** 2018-06-20

**Authors:** Barry Demchak, David Otasek, Alexander R Pico, Gary D Bader, Keiichiro Ono, Brett Settle, Eric Sage, John H Morris, William Longabaugh, Christian Lopes, Michael Kucera, Adam Treister, Benno Schwikowski, Piet Molenaar, Trey Ideker

**Affiliations:** 1 Department of Medicine, University of California, San Diego, La Jolla, CA, 92093, USA; 2 Gladstone Institutes, San Francisco, CA, 95158, USA; 3 The Donnelly Centre, University of Toronto, Toronto, ON, M5S 3E1, Canada; 4 University of California, San Francisco, San Francisco, CA, 94143, USA; 5 Institute for Systems Biology, Seattle, WA, 98109, USA; 6 Inistitut Pasteur, Paris, 75015, France; 7 Amsterdam Medical Centre, Ansterdam, 1105 AZ, Netherlands

**Keywords:** Cytoscape, Automation, App, Network Biology, Network Analysis, Network Visualization

## Abstract

Cytoscape is the premiere platform for interactive analysis, integration and visualization of network data. While Cytoscape itself delivers much basic functionality, it relies on community-written apps to deliver specialized functions and analyses. To date, Cytoscape’s CyREST feature has allowed researchers to write workflows that call basic Cytoscape functions, but provides no access to its high value app-based functions. With Cytoscape Automation, workflows can now call apps that have been upgraded to expose their functionality. This article collection is a resource to assist readers in quickly and economically leveraging such apps in reproducible workflows that scale independently to large data sets and production runs.

## Editorial

Cytoscape is an open source software platform for interactive analysis, integration and visualization of networks and network data ^1^. At heart, Cytoscape provides basic network analysis functionality (e.g., network import/export, network data analysis, visualization and layout) in a menu-driven desktop format. Most importantly, it also enables and encourages users to add extensions (called apps) that deliver custom features important for specific workflows (e.g., ClueGO ^2^ for enrichment analysis relative to various ontologies). To date, Cytoscape users can choose among over 330 apps written by over 550 authors.

In 2014, the CyREST app ^3^ was created to allow external programs to exercise core Cytoscape functionality as part of custom workflows. By authoring such workflows in common languages (such as R and Python), users can combine the best features of Cytoscape with those available in language-specific libraries, thus creating new value much more quickly and cheaply than writing conventional Cytoscape apps. Furthermore, such workflows can more easily integrate external applications (e.g., GenePattern and iGraph) and multiple large datasets. Finally, external workflows enable reproducibility not available using Cytoscape’s standard mouse/keyboard/display interaction mode.

In 2018, Cytoscape Automation was created to enable external workflows to also call functionality in apps. For an app to be callable, it must be upgraded to support automation via either a Commands or Functions interface. As of March, 2018, app authors upgraded and released 22 apps.

This collection of Cytoscape app articles at *F1000Research* is intended to serve as a resource to researchers (as workflow authors) and app developers to understand the features and interfaces exposed by automation-enabled apps. They are written by the app authors themselves according to a template that calls for explaining newly available functions, how they can be called, and with real world examples. We intend that readers be able to quickly and economically incorporate Cytoscape app functionality as reproducible workflows that scale independently to large data sets and production runs.

## Data availability

*No data are associated with this article.*
